# Graph Model-Based Lane-Marking Feature Extraction for Lane Detection

**DOI:** 10.3390/s21134428

**Published:** 2021-06-28

**Authors:** Juhan Yoo, Donghwan Kim

**Affiliations:** 1 Technology Research Team, Incheon International Airport Corporation, Incheon 22382, Korea; juhan.yoo@airport.kr; 2 Center for Intelligent and Interactive Robotics, Korea Institute of Science and Technology, Seoul 02792, Korea

**Keywords:** lane marking detection, line segment, lane marking feature, lane departure warning (LDW) system, lane keeping assist (LKA) system, intelligent vehicle

## Abstract

This paper presents a robust, efficient lane-marking feature extraction method using a graph model-based approach. To extract the features, the proposed hat filter with adaptive sizes is first applied to each row of an input image and local maximum values are extracted from the filter response. The features with the maximum values are fed as nodes to a connected graph structure, and the edges of the graph are constructed using the proposed neighbor searching method. Nodes related to lane-markings are then selected by finding a connected subgraph in the graph. The selected nodes are fitted to line segments as the proposed features of lane-markings. The experimental results show that the proposed method not only yields at least 2.2% better performance compared to the existing methods on the KIST dataset, which includes various types of sensing noise caused by environmental changes, but also improves at least 1.4% better than the previous methods on the Caltech dataset which has been widely used for the comparison of lane marking detection. Furthermore, the proposed lane marking detection runs with an average of 3.3 ms, which is fast enough for real-time applications.

## 1. Introduction

The advanced driver-assistance system (ADAS) helps drivers safely navigate roadways. The rapidly growing smart vehicle industry has prompted ADAS research efforts worldwide [1,2]. Among ADAS systems, the lane-departure warning (LDW) system warns a driver that the vehicle is leaving the host lane when the vehicle is driving above a certain speed [3,4]. In recent years, more vehicles are being equipped with the lane-keeping assist (LKA) system, an extended function of the LDW system. This system combines the LDW function and a function to control vehicle steering. Lane detection, a core technology of these systems, has been researched for many years in the computer vision field [5]. This research can be divided into two categories: neural network-based and hand-crafted-feature-based methods.

The convolutional neural network (CNN) architecture has been employed in various lane detection methods as it continues to substantially achieve better results than previous systems [6,7,8,9,10,11,12,13]. However, this has the disadvantage that the performance is not good enough in other environments that are not included in the training datasets, and CNNs require a considerable amount of data and computational power for the network learning. More specifically, a significant amount of ground truth annotations are required for the learning [14,15]. Furthermore, the analysis is more complex and difficult in neural-network-based methods than hand-crafted-feature-based methods, as a coherent framework for understanding neural-network-based architectures has remained elusive [16]. Therefore, lane detection methods using hand-crafted features have still been actively studied [17,18,19,20,21,22,23,24,25,26,27,28,29].

Lane markings are often damaged by tire abrasion; thus, they may not fully appear in an image [30,31]. Furthermore, various complex road environments make it more difficult to detect lane markings. For lane detection, these conditions can be considered noise, and they tend to have more of an effect on points than lines extracted from an image. As such, using point-based features for lane markings can result in false detection compared to line-based methods [32]. Generally, methods that use features based on lines utilize edges or line segments as the feature [18,19,20,33]. However, as mentioned earlier, lane marking boundaries often become ambiguous due to external influences. This can cause various problems to the line segment extraction: non-extraction or over-extraction. Non-extraction refers to the inability to extract lines from lane markings, and the over-extraction problem involves the extraction of multiple lines from one side of a lane marking.

In this paper, we propose a lane-marking feature extraction method robust to aforementioned problems. We solve the non-extraction problem by extracting the feature points of lane markings for each row of the image and overcome the over-extraction problem by spatially connecting the feature points to each other using a graph structure. Finally, the connected feature points form a line segment.

To accurately extract line segments from the lane markings in road environments where the edges of lane markings are partially damaged, we first introduced a hat filter with adaptive sizes that uses the average intensity of a local region. Note that the image was obtained by a mono-camera installed in the middle of the windshield of a vehicle. The response from the hat filter was used to estimate scores that have high values in regions where real lane markings appear on the road. The local maximum of the scores was extracted as feature points of lane markings by using non-maximum suppression, a low-level computer-vision task. These feature points are then defined as nodes and connected by edges that are generated by the proposed neighbor-searching method. The nodes and edges are used to generate connected graphs, and line segments for lane markings are constructed for each graph. To verify how well the proposed line segments characterize lane markings, we compare the proposed line segments and other ones in terms of lane detection.

Conventional methods generally extract line segments by clustering continuous pixels with similar gradients. However, they cannot often extract line segments when these methods are applied to edges of the lane markings where the partial damage frequently occurs. This can be analyzed for two reasons such as the failure to extract the pixels constituting the line segment and the weak connectivity between the pixels. Eventually, these reasons can yield non-extraction and over-extraction problems, respectably. We propose a new approach robust to these problems, which can extract line segments as features for lane markings by not only using the filter extracting feature points of lane markings for each row of the image but also spatially connecting the feature points to each other by a graph structure.

In a short summary, our contributions are three-fold, as follows: (1) The proposed hat filter with adaptive sizes was applied independently to each row in the image, so that lane-marking feature points that constitute line segments could be extracted even in a small region of the damaged lane markings. Therefore, this suppresses the non-extraction problem as much as possible; (2) The spatial connection information between the feature points can be weakened because they are independently calculated independently for each row in the image. This can cause the over-extraction problem. Thus, we propose a graph model-based lane-marking feature extraction method that can integrate weak spatial connection information efficiently. (3) The proposed final line segment construction method extracts line segments that can better represent the lane markings. The method uses the proposed hat filter response values and a constraint that lane markings should keep a certain width from each other. As a result, outliers can be removed through this process.

This paper is organized as follows. Section 2 discusses related works using the neural network and hand-crafted features in lane detection. In Section 3, the proposed method is introduced. Experimental results are presented in Section 4. Conclusions are given in Section 5.

## 2. Related Work

Traditionally, hand-crafted features have been used for lane detection. Hur et al. [17] demonstrated feature extraction for lane markings using two ridge filters per row to detect multiple lane marks on urban roads. Effectively, their method generates supermarkings, or clusters of neighboring lane-marking features with similar gradient directions. Multiple lane markings are detected using conditional random field graphical models. Notably, the ridge filter [17] is similar to the proposed hat filter with adaptive sizes; however, they use two ridge filters in the right and left directions, whereas we use only one hat filter. Furthermore, it is difficult to estimate the optimal values of user-defined parameters for various environments using the method described.

Aly [34] demonstrated a real-time approach to detect lane markings in urban streets. It first converts an input image into an inverse-perspective-mapping (IPM) image which transforms an image from a camera view to a bird’s eye view by using camera parameters in order to remove the perspective distortion on lane markings. Then, in the IPM image, vertical direction lines were estimated by using a simplified Hough transform and lane markings are detected by utilizing the RANSAC spline fitting method. However, it has disadvantages, such as the fact that it is not applicable to road images with complex environments, and that the IPM accuracy declines if vibrations occur in an image when a vehicle is in motion as in other IPM-based approaches. Gu et al. [35] also used features based on edges to detect lane markings. They extracted lane markings by Hough transform in an edge map obtained by using the canny edge detector. This method can easily and efficiently detect lane markings, however, it is still not sufficiently robust for road images with complex environments.

There are methods that use line segments as a features of lane markings [19,33,36]. They apply the line-segment detector (LSD) [37], because it is robust to noise and has very low computational overhead. However, the LSD can cause false detection for lane markings, as multiple line segments may be extracted from each edge of lane-markings or not be extracted from weak edges (described in further detail in Section 4).

Lee et al. [20] introduced a robust and fast lane detection and tracking method. The method consists of three processes: initialization, detection and tracking. In the initialization process, an input image is first scaled down to a low resolution to reduce computational time. Line segments are then extracted using EDLines [38]. The location of a tentative vanishing point, in terms of lane markings, is calculated based on the distribution of intersection points. By accumulating the vanishing points for a pre-defined number of frames, a valid vanishing point is estimated. In the detection process, the intensities along horizontal lines drawn through the extracted line segments are scanned. Lane-marking candidates are then selected by finding the low–high–low intensity patterns on the horizontal lines. A compact region-of-interest (ROI) is defined based on the accumulation of the lane-marking candidates. Finally, lane markings are detected and tracked in the ROI using a Kalman filter that considers the geometric relationships between the lane-marking candidates and the vanishing point in the tracking process. The authors attempted to achieve maximum performance while satisfying real-time operation. However, their approach still has a limitation that the ROI location cannot be correctly estimated in more diverse and complex road environments.

Jung et al. [39] proposed a lane detector that is robust to short-term noise, such as other road markings, vehicles, shadows and repaired markings, using spatiotemporal images generated by accumulating intensities of rows of pixels with the same position in consecutive frames. In the spatiotemporal image, lane markings are continuously accumulated over the long term, whereas short-term noise quickly disappears. Thus, their method can efficiently remove short-term noise. However, in more complex environments, it can be difficult to align the accumulated lane markings.

Recently, lane detection methods using deep neural networks have shown good performance. Huval et al. [40] proposed a network based on OverFeat framework [41] for lane-marking and vehicle detection using a fully convolutional architecture. The network consists of two branches: binary classification and an integrated regression with seven convolutional layers. This has the disadvantage that it cannot be directly applied to learn a multi-label detector for small objects. Zhu et al. [42] introduced a multi-task network for traffic-sign detection and classification. Their network, which consists of eight convolutional layers, generates a network branch after convolutional layer 6 and it is divided into three branches, while in [40], branching occurs after the layer 7 and it branches into two branches. This allows their network to simultaneously detect and classify traffic signs. Lee et al. [7] was inspired by aforementioned methods, and added a vanishing point estimation branch to three branches of [42], so that their network cannot only detect lane markings and classify road markings but also localize the vanishing point of lane markings.

As discussed earlier, using neural networks for lane detection has several problems. The first one is that its performance declines for environments not included in training datasets. Furthermore, it has limited availability for training datasets of complex road environments and substantial time is required to generate ground truth annotations for training. For these reasons, lane detection methods using the hand-crafted feature have still been studied widely.

## 3. Proposed Method

**Figure 1:**
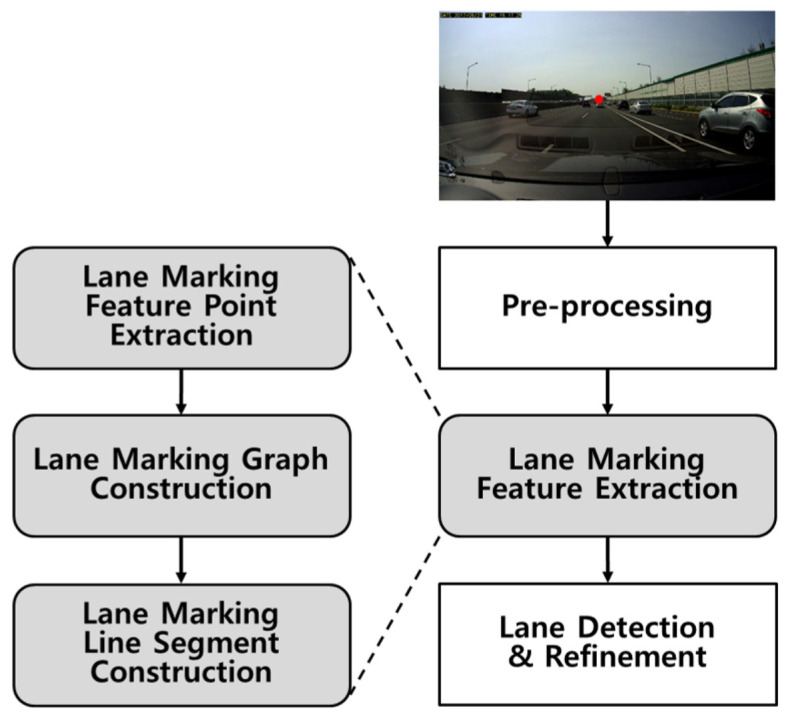
Block diagram of the proposed lane-marking feature extraction and lane detection methods; the image is obtained by a mono-camera. The red point indicates the vanishing point of the lane markings.

The overview of the proposed lane-marking feature extraction and lane detection methods are shown in Figure 1. Once the ROI has been identified and configured, contrast enhancement is performed as a part of the pre-processing step. Using the proposed hat filter with adaptive sizes, lane-marking feature points are extracted and fed as nodes to a graph structure. Then, a line segment is constructed by finding a connected subgraph in the graph. Finally, lane markings are detected and further refined. For an efficient explanation of the proposed graph structure used in this section, various variables are used. Among these, variables for principal components are shown as Table 1.

### 3.1. Region of Interest Construction

An input image is obtained using a mono-camera mounted behind the windshield of a vehicle. Lane markings that are parallel in the real world meet at a vanishing point in the image due to the perspective effect, as shown in Figure 1. This allows the ROI to be defined, as the lane markings are always located in a region below the vanishing point and above the hood of the vehicle. Various methods use a regular or an adaptive ROI based on the vanishing point [20,43,44].

**Figure 2:**
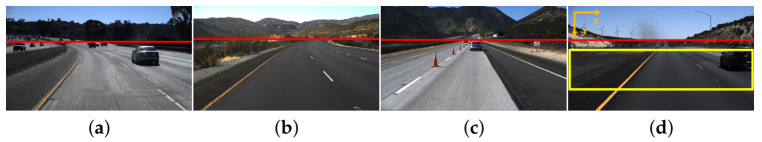
Red lines indicate the vanishing lines of lane markings: (**a**) leftward curve; (**b**) rightward curve; (**c**) downhill; and (**d**) uphill. The yellow rectangle is a regular ROI of the proposed method.

As shown in Figure 2, because LKA or LDW systems are used on high-speed roads, uphill and downhill grades and curved roads are generally constructed with gentle slopes in order to ensure the stability of high-speed driving on the road. Because the input image used for lane detection should include roads located in front of the vehicle, the vanishing line of lane markings is typically located in a row that is higher than a half of the image’s height. Furthermore, the hood of the vehicle is generally located in the region below the image, of which height is about a quarter of the image height in conventional camera installation. Therefore, in order to remove outliers as much as possible, we empirically set the upper and bottom rows of the ROI to a half row of the image and a quarter row of the image height from below the image, respectively. Even if the location of this ROI boundary does not exactly satisfy this constraint, it does not have much effect on extracting the lane-marking features. For example, there are no cases where the vanishing point of lane markings or the road is not visible in the image, although the variation of the road curve is large in our experiments. Furthermore, the proposed method can efficiently estimate lane markings even if a region of the road becomes smaller, because it considers features of the lane markings in each row of the image.

### 3.2. Lane-Marking Feature Point

The color of a lane marking has high contrast with that of the road, so that the driver can see it well. However, the color difference between the lane marking and the road can become diminished if there are changes in illumination, reflections, or shadows, which occur frequently in real road environments. This can increase the false negative rate for lane detection. To reduce this phenomenon, contrast enhancement techniques can be used, such as histogram equalization and gamma correction. In this paper, a histogram equalization approach is used as the pre-processing step, because it is simple and fast to use.

**Figure 3:**
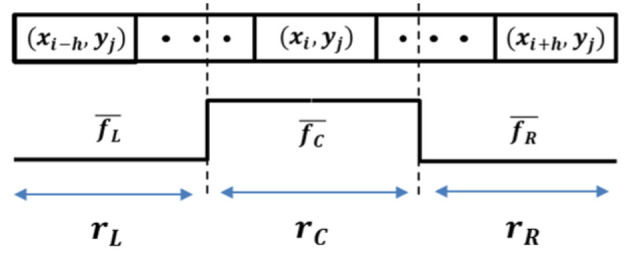
The proposed lane-marking hat filter. $r_{L}$, $r_{C}$, and $r_{R}$ are the size of each region. *i*, *j*, and *h* are indices for the width and the height of the image, and the filter size, respectively. They are satisfying: i−h ≥ 0.

Extractors using positive–negative gradients, hat filters, steerable filters, and methods involving user thresholds can be applied to extract lane-marking features. Of these, the hat filter is intuitive and shows good performance for lane detection [45]. Inspired by this, the proposed approach uses a modified hat filter with adaptive sizes, as shown in Figure 3; this filter, referred to as the *lane-marking hat filter*, consists of three regions: $r_{L}$, $r_{C}$ and $r_{R}$. The size of each region should satisfy the following constraint: $0.5\times r_{C}=r_{L}=r_{R}$. This filter is applied to all pixels, and each pixel to calculate the filter response is centered on $r_{C}$.

The lane-marking hat filter is applied to each row in the image. The maximum filter response value can be expected when the size of $r_{C}$ is equal to the width of the lane marking. However, as shown in the top image in Figure 1, lane-marking widths in each row have different sizes; the lane-marking width is the widest at the bottom row of the image. Thus, it is necessary to estimate an appropriate width for each row to attain the maximum response value of the filter. In order to do this, the lane-marking hat filter has an adaptive size for each row. The lane-marking width becomes linearly thinner as it approaches its vanishing point due to the perspective effect in the image. Therefore, if the maximum and minimum values of the lane-marking width in the image are known, the lane-marking width for each row can estimated by using a simple proportional formula. In other words, the maximum size of the lane-marking hat filter corresponds to the maximum size of the lane-marking width at the bottom row of the image, and the filter size decreases for each row by the following *step size* as the row goes to the upper row of the image: the *step size* = $(\alpha_{max}-\alpha_{min})/\beta$, where $\alpha_{max}$ and $\alpha_{min}$ are the maximum and minimum value of the lane-marking width, respectively. β is the size of the ROI height. In our experiments, typical values for $\alpha_{min}$ and $\alpha_{max}$ are 3 and 15 pixels, respectively.

Let $I(x_{i},y_{j})$ denote the intensity at $\left(x_{i},y_{j}\right)$ in the histogram-equalized image, where *i* and *j* are indices of the width and the height of the image, respectively. Let $N_{C}$, $N_{L}$, and $N_{R}$ be the number of pixels in the lane-marking hat filter regions, $r_{C}$, $r_{L}$, and $r_{R}$, respectively. The response $\gamma_{ij}$ of the lane-marking hat filter at all pixel locations in the input image Ω, $\Omega ={\left\{\left(x_{i},y_{j},\gamma_{ij}\right)\right\}}^{M}$, where *M* is the number of pixels, is calculated as follows:

(1) $$\gamma_{ij}=\;\left\{\begin{array}{c} \begin{array}{cc} 2\cdot \bar{f_{C}}\left(x_{i},y_{j}\right)-\bar{f_{R}}\left(x_{i},y_{j}\right)-\bar{f_{L}}\left(x_{i},y_{j}\right), & \bar{f_{C}}>\bar{f_{L}}\;and\;\;\bar{f_{C}}>\bar{f_{R}}\\ 0, & otherwise \end{array} \end{array}\right.,$$

where:

$$\begin{array}{c} \bar{f_{C}}\left(x_{i},y_{j}\right)=\sum_{\left(x_{i},y_{j}\right)\in r_{C}}I\left(x_{i},y_{j}\right)/N_{C},\\ \bar{f_{L}}\left(x_{i},y_{j}\right)=\sum_{\left(x_{i},y_{j}\right)\in r_{L}}I\left(x_{i},y_{j}\right)/N_{L},\\ \bar{f_{R}}\left(x_{i},y_{j}\right)=\sum_{\left(x_{i},y_{j}\right)\in r_{R}}I\left(x_{i},y_{j}\right)/N_{R}. \end{array}$$

**Figure 4:**
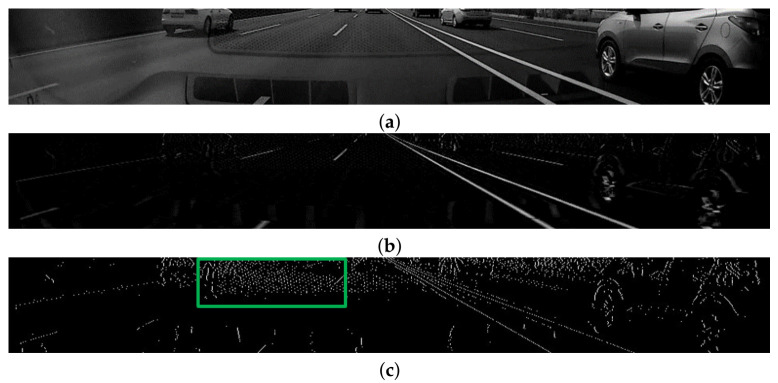
Examples for lane-marking feature points extraction: (**a**) ROI image; (**b**) normalized lane-marking scores image; and (**c**) lane-marking feature points image.

We define $\gamma_{ij}$ as the *lane-marking score*; a high value corresponds to where lane markings exist. In Figure 4b, white points represent normalized lane-marking scores; the higher the score, the brighter the color. From the image, we can see that the proposed lane-marking hat filter yields responses with high scores from lane markings. Finally, the local maximum of $\gamma_{ij}$ is extracted using standard non-maximum suppression. Then, pixels at $\left(x_{i},y_{j}\right)$ with low lane-marking scores within the lane-marking width can be removed. As shown in Figure 4c, the maximum point is defined as the *lane-marking feature point* $p_{k}$, $P={\left\{p_{k}\left(x_{i},y_{j},\gamma_{ij}\right)\right\}}_{k=1}^{K}$, K= ∣P∣, K<M, and P⊂Ω. For notation brevity, let *k* denote a pair of *i* and *j* indices. This process is performed for each row, similar to the lane-marking hat filter process, and its kernel size corresponds to the maximum lane-marking width.

### 3.3. Lane-Marking Line Segment

In this paper, we propose the *lane-marking line segment* (**LaLi**) as a feature to detect lane markings. This is extracted through several steps as follows. A connected graph is constructed by grouping $p_{k}$ whose geometric distances are close to each other. Then, a connected subgraph with a maximum lane-marking score is extracted from the graph. A LaLi is constructed by fitting the extracted subgraph to a line segment. There are studies using graph models [13,17,46]. As mentioned in Section 2, Hur et al. [17] used a conditional random field graphical model to detect multiple lane markings. Lu et al. [13] first extracted low-level features by using a hierarchical semantic segmentation network. Then, the features are fed to a graph by considering geometric prior and topology. In [46], lane marking features are extracted by utilizing a cascade lane features detector and they are fed to a weighted graph in which the weight is corresponding to confidence of pixels to be lane points. Finally, lane markings are estimated by using a particle filter.

#### 3.3.1. Lane-Marking Graph

**Figure 5:**
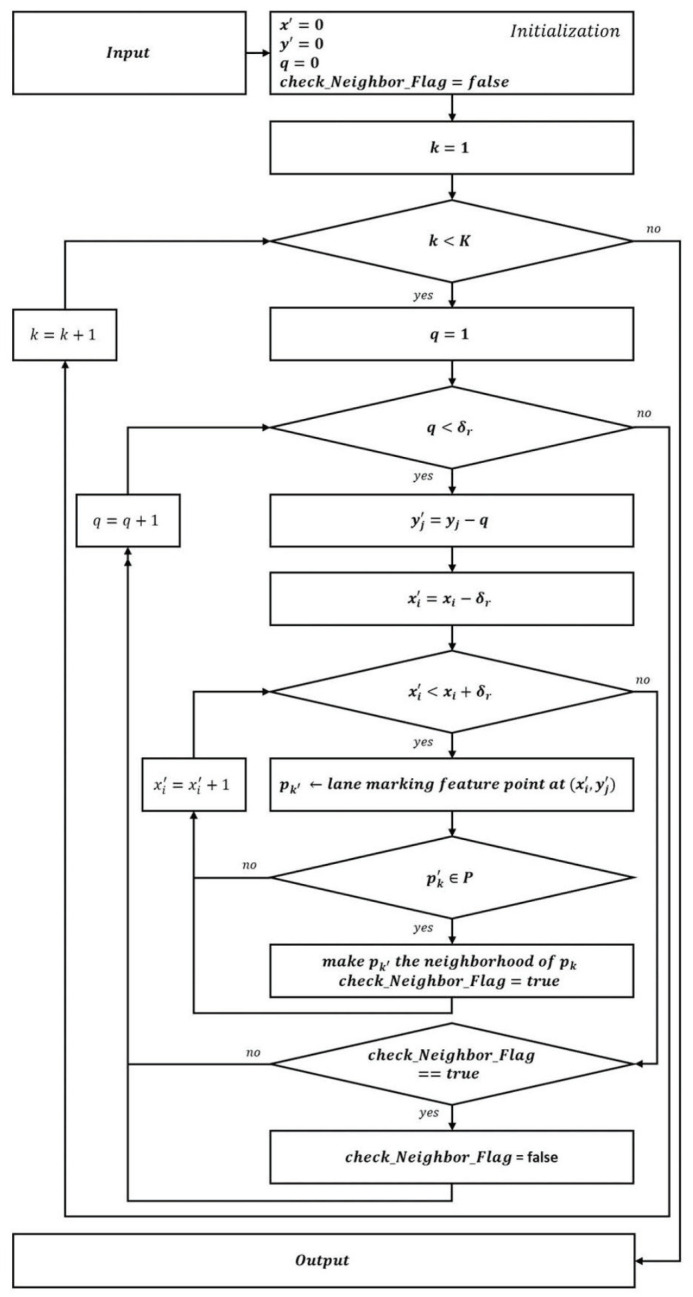
Diagram for finding neighbors.

Let graph G=(P,E) defined as $G=\{g^{1}\cup g^{2},\cdots ,\cup g^{t},\cdots ,\cup g^{T}\}$, where $g^{t}=\left(P^{t},E^{t}\right)$ be an undirected subgraph of *G*, called *lane-marking graph*. In here, *P* is a set of nodes in *G* and the edge set *E* is satisfies E⊂P×P. $P^{t}$ is a set of nodes in the *t*-th lane-marking graph and satisfying $P^{t}\subset P$. Similarly, $E^{t}$ is a set of edges in *t*-th lane-marking graph and satisfying $E^{t}\subset E$. Here, $P^{t}={\left\{p_{v}^{t}\right\}}_{v=1}^{V^{t}}$, *v* is a set of indices for nodes in $g^{t}$ ($v=1,2,\cdots ,V^{t}$), and $E^{t}={\left\{e_{u}^{t}\right\}}_{u=1}^{U^{t}}$, *u* is a set of indices for edges in $g^{t}$ ($u=1,2,\cdots ,U^{t}$). An edge $e_{u}^{t}=\left(p_{v1}^{t},p_{v2}^{t}\right)$ is incident with $p_{v1}^{t}$ and $p_{v2}^{t}$, where v1, v2∈v and v1≠v2, in which they should be neighbors, as shown in Figure 5. Note that $p_{v}^{t}$ with the lowest *y* coordinate in the image has no neighbors because it cannot find other nodes. Furthermore, $\delta_{r}$ presents the range of finding neighbors and its value empirically set to the maximum lane-marking width in all the experiments. In Figure 5, **Input**: $p_{k}\in P$ and ∣P∣ =K, $\delta_{r}$ = a user threshold for the range to find the neighbor, and ($x_{i},y_{j}$) is the coordinate of $p_{k}$. **Output**: verify whether or not the $p_{k}$ has neighbors.

In this neighbor relationship, $p_{k}$ is defined as the parent and its neighbors as children; one child can have several parents. Then, the lane-marking graph is similar to a tree but is not a tree, and becomes a connected graph despite the removal of any edge. Furthermore, there can be $p_{k}$ that has no children or has never become a child. We call these the *leaf* and the *root*, respectively. In this case, a lane-marking graph consists of one root and at least one leaf, and it is a connected graph.

The lane markings in the lower region appear thicker and more clearly than those in the higher region in the image. Therefore, $p_{k}$ in the lower region where lane markings exist can have high lane-marking scores. If the $p_{k}$ is selected among them as the root in a lane-marking graph, the graph may contain many $p_{k}$, which have a high probability of being lane markings. Thereby, LaLis, which represent the lane markings, can be efficiently constructed. To do so, $p_{k}$ is sorted in descending order by the *y* coordinate before the lane-marking graph is constructed. We then search the roots in the sorted *P* and construct lane-marking graphs based on each root. Note that $p_{k}$, except for the root, can be shared in each graph.

#### 3.3.2. Lane-Marking Line Segment Construction

**Figure 6:**
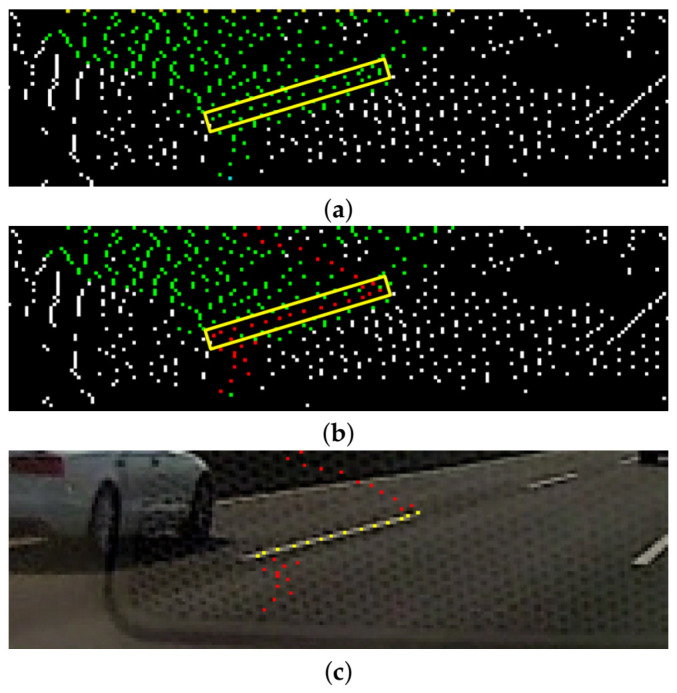
Examples of lane-marking line segments construction: (**a**) white and green points represent $p_{k}$ and $p_{v}^{t}$, respectively. Yellow points (from top row) and one cyan point (from bottom row) denote leaf nodes and a root node of the $g^{t}$, respectively. A yellow rectangle depicts a region where a real lane marking exists; (**b**) an optimal path corresponding to $g_{opt}^{t}$ is marked with red points; and (**c**) points that make up a LaLi are depicted as yellow points on the real input image.

$g^{t}$ can include $p_{v}^{t}$ extracted from a non-lane-marking region due to noise. These nodes $p_{n}^{t}$ may prevent the precise extraction of LaLis: here, $P_{neg}^{t}={\left\{p_{n}^{t}\right\}}^{N^{t}}$, n∈v, $N^{t}<V^{t}$, and $P_{neg}^{t}\subset P^{t}$. Therefore, they are removed from the $g^{t}$ by using lane-marking scores. Note that $p_{v}^{t}\in \left\{P^{t}\setminus P_{neg}^{t}\right\}$ should be connected. Eventually, the problem can evolve into one attempting to find the connected optimal subgraph $g_{opt}^{t}$ in $g^{t}$, where $g_{opt}^{t}$ is subject to the following constraints: $g_{opt}^{t}\subset g^{t}$, $g_{opt}^{t}=\left(P_{opt}^{t},E_{opt}^{t}\right)$, $P_{opt}^{t}={\left\{p_{o}^{t}\right\}}_{o=1}^{O^{t}}$, o∈v, $O^{t}<V^{t}$, $P_{neg}^{t}\cup P_{opt}^{t}=P^{t}$, and $P_{neg}^{t}\cap P_{opt}^{t}=\phi$. To sum up, as shown in Figure 6b, $p_{o}^{t}$ and $p_{n}^{t}$ are represented as red and green points, respectively. When all the red and green points are summed together, they represent $p_{v}^{t}$.

In this paper, to find $g_{opt}^{t}$, only paths between one root and individual leaves are considered, as opposed to paths between all pairs of $p_{v}^{t}$. Out of these paths, the optimal path in which the lane-marking score of the sum of the nodes is the maximum is selected as the $g_{opt}^{t}$. We find this path using the Dijkstra algorithm, which is a general method in graph theory used to find the optimal path between two nodes. Figure 6b shows an example of finding the optimal path using the proposed method. The optimal path includes many $p_{v}^{t}$ with a high probability of being lane markings and is extracted as a subgraph that consists of interconnected nodes.

The LaLis can now be extracted from the $g_{opt}^{t}$. Han et al. [47] introduced a curvature extraction method with low computational power; and the proposed LaLi extraction technique drew inspiration from this method. For all pairs of nodes $\left(p_{o1}^{t},p_{o2}^{t}\right)\in P_{opt}^{t}$, where o1,o2∈o, and o1≠o2, we consider a line segment $\ell_{o1,o2}^{t}$ that has these two nodes as endpoints. By considering the shortest distance between $\ell_{o1,o2}^{t}$ and $p_{\tilde{o}}^{t}$ in $g^{t}$, where $\tilde{o}\in o$ and $\tilde{o}\notin \left(o1,o2\right)$, the sum of lane-marking scores *F* is defined as follows:

(2) $$F_{o1,o2}^{t}=\sum_{\begin{array}{c} \tilde{o}\in o\\ \tilde{o}\notin o1,o2 \end{array}}\gamma_{\tilde{o}}^{t},$$

subject to:

(3) $$\begin{array}{c} Distance\left(p_{\tilde{o}}^{t},\ell_{o1,o2}^{t}\right)<\delta_{d},\\ Length\left(\ell_{q1,q2}\right)>\delta_{l}, \end{array}$$

where $\gamma_{\tilde{o}}^{t}$ is the lane-marking score at $p_{\tilde{o}}^{t}$. Distance() and Length() are the shortest distance between $p_{\tilde{o}}^{t}$ and $\ell_{o1,o2}^{t}$, and the length of $\ell_{o1,o2}^{t}$, respectively. $\delta_{d}$ and $\delta_{l}$ are user thresholds. We then identify two indices, o1 and o2, in which *F* is the maximum:

(4) $$L_{o1,o2}^{t}=\underset{o1,o2\in o}{\arg\max}\;F_{o1,o2}^{t}.$$

A line segment with two end points, $p_{o1}^{t}$ and $p_{o2}^{t}$, is constructed as a LaLi. Figure 6c shows a sample result of an extracted $L_{o1,o2}^{t}$ from the $g^{t}$; as shown in the figure, the proposed method correctly extracts a line segment from the $g^{t}$.

Multiple LaLis can be extracted from a region, including $p_{k}$ with high lane-marking scores, because $p_{k}$, except for the root, can be duplicated in different $g^{t}$. In this case, a LaLi with a maximum *F* is selected from the LaLis in the region. Furthermore, multiple LaLis can be extracted from a solid or a long broken lane marking in the case of lane-marking damage caused by heavy traffic or wear. Therefore, the extracted LaLis are merged by using the following constraints: the distance between endpoints of each LaLis is short (<$\delta_{e}$) and the difference in the gradients between LaLis is small (<$\delta_{g}$).

In order to find an optimal path of the proposed graph, an energy that considers both the lane-marking scores and the geometric relationships between $p_{k}$ can be considered. This energy can be applied as a weight to the node or edge of the graph. Then, the optimal path finding problem can convert the optimal path finding problem into a combinatorial optimization problem that minimizes the energy and it can be solved by using dynamic programming [48] without using the several steps mentioned above. However, this approach can cause fragmentary LaLis because the graph can be disconnected by peak noises. In contrast, the proposed method prevents LaLi fragmentation, as it guarantees that all $p_{k}$ are connected.

#### 3.3.3. Lane-Marking Line Segment Score Updating

If the lane markings are parallel, the distance between them should remain constant in each consecutive frame. This can be used as a geometric constraint for lane detection. In general, lane markings do not appear parallel in images obtained from the camera view, due to the perspective effect. As mentioned in Section 2, using the IPM method makes it easier to use the constraint because lane markings appear in parallel in the transformed image [49,50]. However, the effectiveness of the IPM can be reduced by incorrect mapping in dynamic road environments. Therefore, to remove the outliers, we apply the constraint directly to the image obtained from the camera view, without using the IPM.

**Figure 7:**
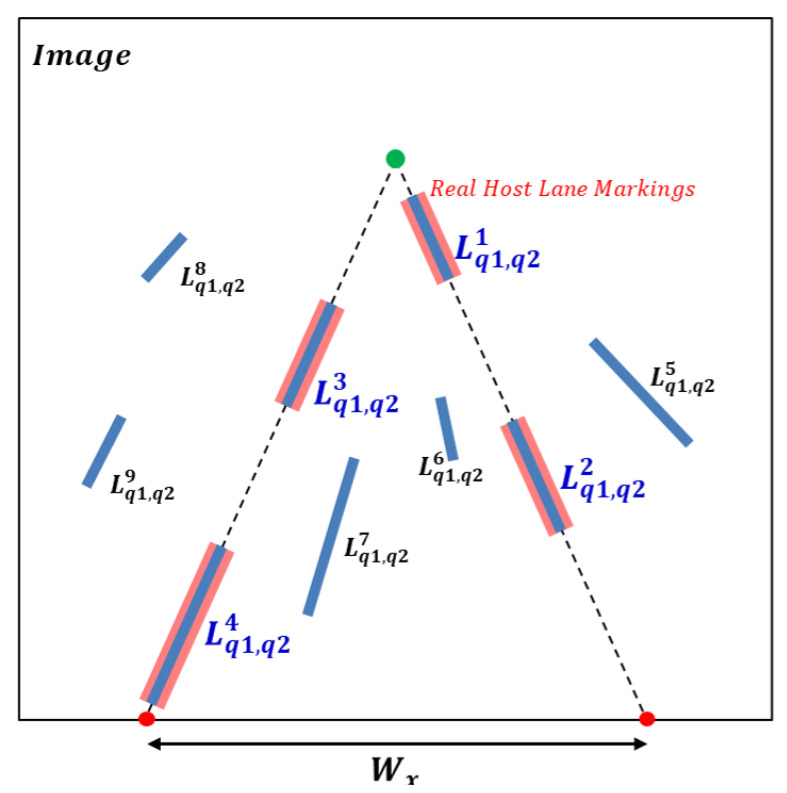
Example of lane-marking line segment score updating. Red rectangles are real broken host lane markings and constructed LaLis are represented as blue rectangles. The green point is the vanishing point of the host lane markings. Two cross points between the lower boundary of the image and extended host lane markings (dotted lines) are represented as red points.

Lane markings generally consist of solid and broken lines. Left and right solid lane markings are symmetric with respect to a vertical line passing through the vanishing point of the host lane markings at the same horizontal position. However, broken lane markings may not be symmetrical, as shown in Figure 7. To enforce a constraint that the lane width should be kept constant, a left LaLi tilted to the left and a right LaLi tilted to the right are first selected. The left and right LaLis are extended to the lower boundary of the image, and then two intersection points are obtained on the boundary. We consider the distance between the two intersection points as the host lane width, $W_{x}$, with the condition: $\delta_{down}<W_{x}<\delta_{up}$, where $\delta_{down}$ and $\delta_{up}$ are user thresholds. If the left LaLi has a right LaLi satisfying the $W_{x}$ condition, a weight is added to the score of the left LaLi. This process is repeated for the right LaLi.

### 3.4. Lane Detection and Refinement

Lines that are parallel in the real world meet at a vanishing point in an image. Conversely, the point can be used to find lines that are parallel. Given that LaLis are designed to be extracted mainly from lane markings and that they also satisfy this property, their convergence point has a high probability of being the vanishing point of the lane markings. By estimating the vanishing point, it is possible to efficiently remove the outliers included in LaLis, thus improving lane detection.

Therefore, we use [19] in order to correctly estimate the vanishing point. This method can estimate the vanishing point of lane markings by using the voting function that is defined with line segment strength that represents the relevance of the extracted line segments. Furthermore, it provides the efficient lane detection method utilizing not only the geometric relationships between the line segments and the estimated vanishing point, but also the inter-frame similarity in consecutive frames. We modified the vanishing point estimation approach by replacing the proposed LaLis with the line segments in order to correctly estimate the vanishing point of lane markings from a noisy image, and apply the lane detection method to determine lane markings. Note that the line segment strength should be modified to suit the proposed LaLi and it is defined as the lane-marking score *F* in the proposed method. With the probabilistic voting framework [19], sensing noise in the imaging process can be efficiently handled, and more robust lane detection can be obtained. Our experimental results show that the proposed LaLi was well applied to the framework and performed better than using line segments extracted by the LSD for lane detection.

## 4. Experimental Results

By analyzing the results of the lane detection method using the proposed features, we verify how well the proposed features characterize lane markings. There are large datasets such as the *KITTI* benchmark [51] that include various fields, and the recently introduced *tusimple* dataset [52] for the lane detection challenge. However, *KITTI* does not have ground truth for each lane markings, and *tusimple* is not currently available. Therefore, we utilized two other datasets containing the ground truth for each lane markings for evaluating lane detection. In order to demonstrate the robustness of the proposed feature based on the line segment, we used a lane detection method that utilizes line segments extracted by another popular line segment extractor as features of lane markings. The proposed features are applied to the method instead of the line segments, and then we compare lane detection results by the method.

One is the *Caltech dataset* [34], which has been used in numerous studies [7,19,33,49,53] for the lane detection. As shown in Figure 10, the dataset consists of images obtained from four environments during the daytime: cordova1, cordova2, washington1, and washington2 (from top to bottom). It consists of 250, 406, 337 and 232 images, respectively. Lane markings in images in the dataset are partially covered by shadows but clean overall. Therefore, this dataset has the disadvantage of lacking a variety of road environments.

**Figure 8:**
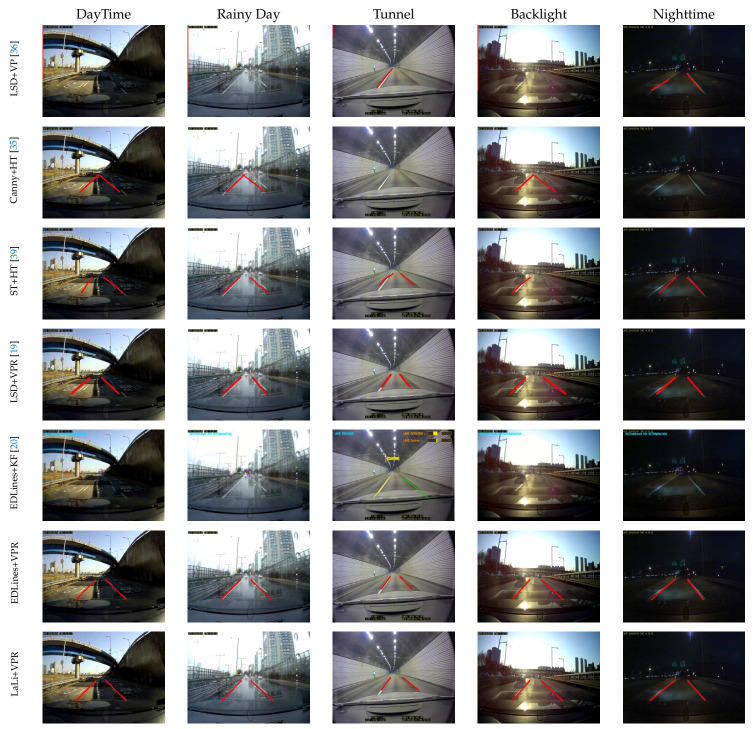
Sample lane detection results on KIST dataset [19]. LaLi+VPR is the lane detection method using the proposed lane-marking features.

The other is the *KIST dataset* [19], which consists of images acquired from more diverse environments than in the Caltech dataset, such as illumination changes, weather changes, and roads having lane markings that are eroded or damaged by traffic. As shown in Figure 8, it consists of five environment sequences: daytime, a rainy day, a tunnel, backlight conditions, and nighttime (from top to bottom). They are acquired by a front-mounted camera in the car on arterial roads and the numbers of images are 525, 222, 736, 359, and 263, respectively.

In this paper, we use these two datasets to test the performance of the proposed lane detection method using LaLis and to compare the method with other approaches. The detection criteria introduced in [19] are used to determine the lane detection accuracy as follows: in an image, if minimum and median distances between all the points on the detected and ground-truth lane markings are closer than 5 pixels, respectively, we determine it as the correct detection and it is performed for the left and right lane markings, respectively. Finally, left, right, and total lane detection rates are $m^{l}/Z$, $m^{r}/Z$, and $(m^{l}+m^{r})/2Z$, respectively. *Z* is the total number of images. All the experiments are implemented in C++ and run on a PC with 64G RAM and a 4Ghz CPU using a single thread. The parameter values used in this experiment are $\delta_{d}=1$, $\delta_{l}=0$, $\delta_{e}=3$, $\delta_{g}=5$, $\delta_{down}=110$, and $\delta_{up}=140$.

### 4.1. Lane Detection on Kist Dataset

We compare our proposed method with five baseline methods that have four different lane-marking feature types. Liu et al. [36] used line segments extracted by LSD [37] as the lane-marking feature and lane markings are detected by utilizing the *vanishing point* (**VP**) of the line segments. Similar to [36], Yoo et al. [19] also used the line segments extracted by LSD and the vanishing point of them, however, their method differs to [36] in that it improves the accuracy of the *vanishing point location by considering the relevance* (**VPR**) of the line segments. Lee et al. [20] utilized line segments extracted by EDLines [38] and lane markings are detected and tracked by using the *Kalman filter* (**KF**). Gu et al. [35] used edges extracted by the *canny edge detector* (**Canny**) and lane markings were detected from the lines extracted by the *Hough transform* (**HT**). Jung et al. [39] utilized the *spatiotemporal image* (**ST**) to extract line-marking features and detect the lane markings by applying the Hough transform. These baseline methods are referred to as LSD+VP [36], LSD+VPR [19], EDLines+KF [20], Canny+HT [35] and ST+HT [39], respectively. Additionally, we test a method using line segments extracted by EDLines [38] as the lane-marking features and the lane detection approach of [19], called EDLines+VPR.

As shown in Figure 8, we compared the sample results of the proposed method with these baseline methods (from left to right); the lane detection method using the proposed lane-marking features is referred to as LaLi+VPR. The detected lane markings are displayed as red lines in the figure; in the result images using EDLines+KF, the detected lane markings are represented by green and yellow lines, and the estimated vanishing point of the lane markings is shown as a red cross. Overall, LaLi+VPR showed good lane detection performance. The sample results of LaLi+VPR, LSD+VPR, and EDLines+VPR detect lane markings well based on the lane detection criteria. However, LaLi+VPR detects the position of lane markings more accurately.

Table 2 shows the lane detection accuracy on KIST dataset, in which accuracy is considered separately for the left, right and both lane markings; the best accuracy is represented in blue, green and red, respectively. LSD+VP calculates a mean of intensity values from each row, and then the row with the minimum mean value is selected as the upper boundary of the ROI image. However, their method may not work well in diverse road environments, such as on KIST dataset. Therefore, we modified their method by changing their ROI to the fixed ROI used in LaLi+VPR, and then the lane detection accuracy is calculated from the modified LSD+VP. Canny+HT quickly detects lane markings using the simple properties of lane markings, such as the brightness difference between lane markings and the road, certain angles of lane markings that appear in the image, and so on. However, similar to LSD+VP, it has the disadvantage that lane-marking locations cannot be accurately estimated for variable road conditions. Furthermore, the approach using edges extracted by Canny or Sobel edge detectors can extract a lot of edges that are not related to lane markings so that it can cause many false lane detections. Therefore, on the KIST dataset, the overall lane detection accuracies of the aforementioned methods were low, whereas those of ST+HT were higher than those of other methods due to the advantage of having the spatiotemporal image. However, if left-right movements of the vehicle or the variation in the lane width are large, the lane detection accuracy can be lowered because it is difficult to align the lane markings in the spatiotemporal image. The night sequence includes an interchange section and a large variation of the lane width. Therefore, in the sequence, the lane detection accuracy of ST+HT was low, whereas LSD+VPR and LaLi+VPR show good performance.

In the tunnel sequence, the lane width remains nearly constant, but sections in which the lane markings are difficult to distinguish from the road are included. While LSD+VPR, EDLines+VPR, and LaLi+VPR utilize a small number of frames obtained in the short-term to detect and refine lane markings, ST+HT detects lane markings using the spatiotemporal image, consisting of frames obtained over a longer period. Therefore, ST+HT was able to accurately identify the lane markings, even in the sections where it was difficult to see the lane markings for the long period. Their method outperformed most other methods in the tunnel sequence; however, LaLi+VPR was compatible with it, in terms of the sequence.

As mentioned in Section 2, EDLines+KF uses accumulated results to improve lane detection accuracy. The accumulation is performed for a certain number of frames in each of three processes: vanishing point estimation, compact ROI determination and tracking. The processes are performed step by step, and their entire framework is restarted from the beginning if even one of the processes fails. Thus, this method may accumulate many frames before the lane markings are detected and tracked exactly. For this reason, EDLines+KF did not show good performance on the KIST dataset, as the dataset includes many images with external environmental noise, such as shadows and road markings. Notably, the rain sequence consists of 222 images, the smallest number of images among other sequences. Because the number of images is insufficient for their method to obtain detected results with high reliability, their method is not available for the rain sequence. Images in the tunnel sequence have a smaller variation in lane width than those of other sequences. Therefore, their method showed better performance in the tunnel sequence than in other sequences.

For all the sequences, the lane detection accuracies of LaLi+VPR were higher than those of LSD+VPR and EDLines+KF. This indicates that the proposed LaLi is not only a better representative of real lane markings than the line segments extracted by the LSD and EDLines but also can help to detect the lane markings more accurately.

**Figure 9:**
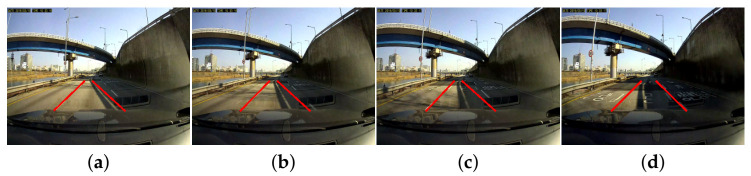
Sample result images of false detection in consecutive frames in the daytime sequence on KIST dataset: (**a**) T frame; (**b**) T+1 frame; (**c**) T+2 frame; and (**d**) T+3 frame.

Figure 9 shows the sample result images of false detection of LaLi+VPR in consecutive frames. The right-side lane marking was detected incorrectly at frame T because the long bright region between shadows had not only a width but also a gradient similar to those of the real right lane marking, although LaLis are extracted on all real lane markings in these images. Furthermore, the lane marking was not accurately detected up to frame T+2 because the refinement method of [19] is used. However, it was detected correctly at frame T+3; the accuracy here was dependent on the parameters of the refinement method.

In order to verify running time, we considered the average time taken to extract the proposed LaLis from each image for all the images in the KIST dataset. We also measured the same average time for LSD, which has a very fast running time, and compared it with the result of LaLi. The LSD and LaLi took an average of 1.4 ms and 3.3 ms, respectively. Although the LSD was approximately 2 ms faster than LaLi in terms of average time, the proposed method was sufficient to run in real time.

### 4.2. Lane Detection on Caltech Dataset

**Figure 10:**
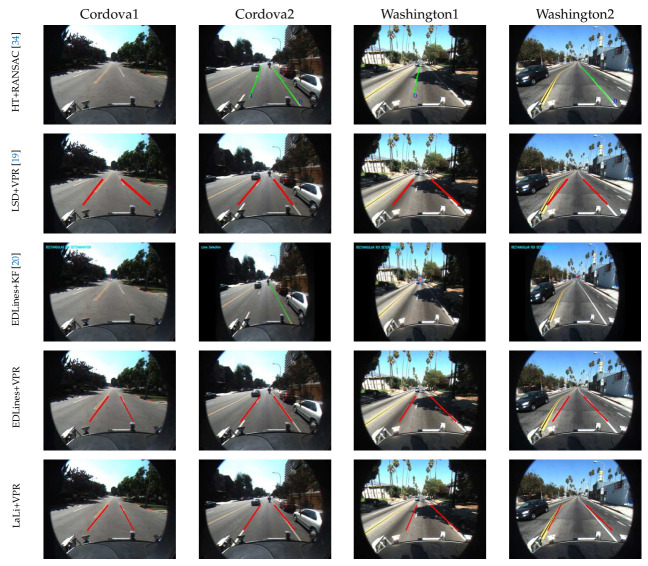
Sample lane detection results on Caltech dataset [34].

In Figure 10, the sample shows results of lane detection on the Caltech dataset in which three baseline methods are compared to LaLi+VPR (from top to bottom): Aly’s method [34], LSD+VPR [19], EDLines+KF [20], EDLines+VPR and LaLi+VPR. LSD+VPR, EDLines+KF and EDLines+VPR are the aforementioned methods, and Aly’s method [34] uses the simplified Hough transform and then lane markings are detected by utilizing the RANSAC spline fitting (referred to as **HT+RANSAC**).

Note that the washington1 sequence of the Caltech dataset includes not only images in which the right lane markings do not clearly appear but also a lane-change section. Lane markings detected by using HT+RANSAC and EDLines+KF are represented as green lines. Red lines indicate lane markings detected by using LSD+VPR, EDLines+VPR and LaLi+VPR. In the sample result images using EDLines+KF, the detected lane markings and the estimated vanishing point of lane markings are represented by green lines and a red cross, respectively.

In the sample result images of cordova1, HT+RANSAC did not detect any lane markings, and LSD+VPR did not detect the right lane marking correctly, whereas LaLi+VPR and EDLines+VPR accurately detected both right and left lane markings. With the exception of LaLi+VPR and EDLines+VPR, the other methods did not detect the left lane marking correctly, as shown in the sample result images of cordova2. However, LaLi+VPR detected the left lane marking more accurately than EDLines+VPR. In the sample result images of washington1, HT+RANSAC detected only the left lane marking, whereas LSD+VPR, EDLines+VPR and LaLi+VPR detected both the right and left lane markings. However, LaLi+VPR detected the left lane marking closer to its ground truth position than did that of LSD+VPR and EDLines+VPR. The sample result images of washington2 show that LaLi+VPR accurately detected the lane markings, although the width of the host lane was not constant.

As mentioned earlier, EDLines+KF requires a significant number of accumulated detection results to increase the reliability of the lane detection and tracking. There are fewer images in cordova1 and washington2 than in the other sequences. Therefore, the lane detection accuracies of EDLines+KF were lower in these sequences than in other sequences. The Caltech dataset includes many sections in which lane markings do not appear, e.g., crosswalks and crossroads. As this can prevent the sufficient accumulation of detected results with high reliability, the accuracy of EDLines+KF was lower than that of other methods.

Lane detection accuracies, computed based on the detection criteria introduced in [19], are summarized in Table 3. In the washington1 sequence, the lane detection accuracy of LaLi+VPR was lower than that of EDLines+VPR because false detection occurred while changing lanes. However, LaLi+VPR is compatible with EDLines+VPR. Overall, the lane detection accuracies of LaLi+VPR were higher than those of LSD+VPR and EDLines+VPR. On the washington1 sequence, although the accuracy of LaLi+VPR slightly lower than EDLines+VPR, it achieves the comparable result. From the results of LSD+VPR, EDLines+VPR, and LaLi+VPR, we can see that the proposed LaLis were more representative of the real lane markings than line segments extracted by the LSD and EDLines.

### 4.3. Lane Marking Feature Extraction

**Figure 11:**
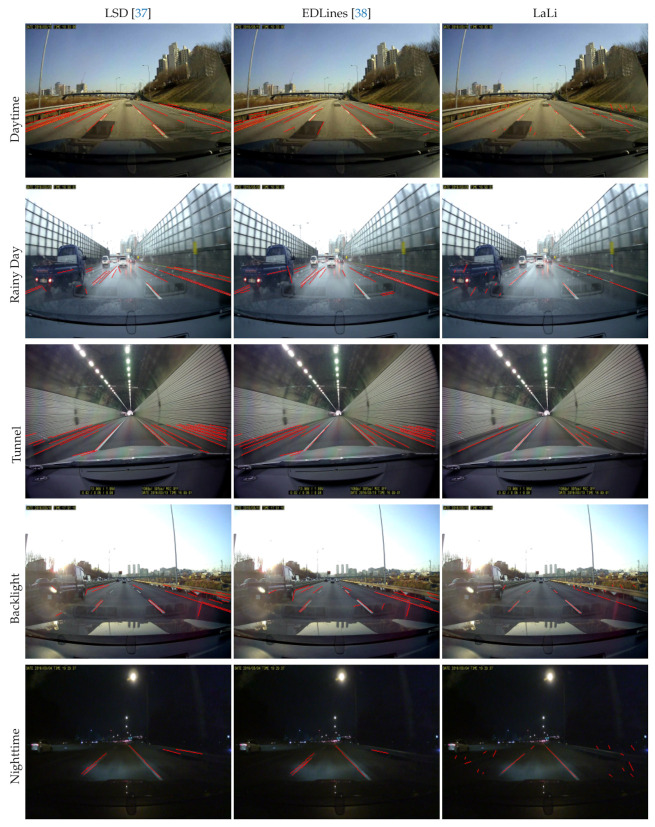
Sample results of line segment extraction on KIST dataset.

We compare LaLi with line segments extracted by LSD [37] and EDLines [38] that are used as features of lane markings for the lane detection on the KIST dataset. Figure 11 shows the sample results of line segments extracted by LSD, EDLines, and the proposed LaLi extraction method (from left to right).

A real lane marking was painted as a bright line having a constant width for good visibility, such that more than two edges could be extracted from its boundary. Therefore, overall, the LSD and EDLines extracted two line segments on opposite sides of one lane marking, and this could yield false lane detection. However, the proposed method extracted one line segment exactly at the center of the lane marking.

The LSD and EDLines determined whether a line segment, which is represented by a single rectangle, is validated in the image using a contrarian approach [54,55]; often, it cannot extract the line segments of real lane markings although the markings are clearly visible, as shown in the left-top lane marking in the host lane during the daytime, backlight, and nighttime sequences. In contrast, the proposed LaLis were accurately extracted on these sequences.

Furthermore, as shown in Table 2 and Table 3, LaLi+VPR outperformed LSD+VPR and EDLines+VPR on almost all datasets except for the washington1 sequence. This shows that the proposed LaLis represent lane markings better than line segments extracted by the LSD and EDLines. Note that LaLi+VPR, EDLines+VPR, and LSD+VPR have the same lane detection method but they use different line segments as the lane-marking features.

The proposed method, LSD, and EDLines extracted about 20.59, 22.60, and 22.21 line segments on average per image, respectively. Eventually, this also shows that LaLi can help to more accurately detect lane markings with lesser line segments than LSD and EDLines.

The proposed method can extract line segments that are robust to non-extraction and over-extraction for lane markings with a constant thickness of at least 3 pixels, as mentioned in Section 3. However, as shown in LaLi results on the daytime, rainy day, and backlight sequences in Figure 11, the rightmost lane markings on the road have a thin thickness: one to two pixels in the image. Therefore, in these cases, the proposed method extracted several LaLis on the lane marking since the widths of the lane markings are too thin.

### 4.4. Comparison with Neural-Network-Based Methods

**Figure 12:**
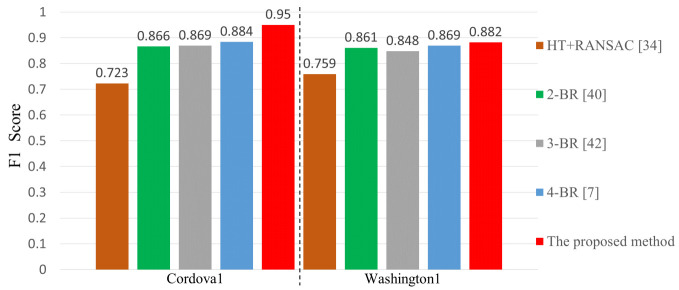
Comparison of neural-network-based methods and the proposed method.

Figure 12 shows a comparison of the F1 score results of LaLi+VPR, HT+RANSAC [34], and neural-network-based methods [7,40,42] on the Caltech dataset. As mentioned in Section 2, the neural-network-based methods use a similar neural-network structure, but they have a different number of branches; we refer to them as **2-BR** [40], **3-BR** [42] and **4-BR** [7], respectively. As shown in Figure 10, images of the dataset have clear lane markings and uncomplicated environments. On the cordova1 sequence, the proposed hand-crafted-feature-based method is 0.066 higher than the best performing method 4-BR among the neural-network-based methods. The washington1 sequence has a lot of shadows of trees than the cordova1 sequence. On the sequence, apart from HT+RANSAC, the performance of the other methods is similar, but the proposed method is the best.

4-BR used the datasets consisted of about 20,000 images with 17 lanes and road markings classes, and introduced a robust and fast lane detection method using CNNs. However, their method has lower performance than the proposed method on images with uncomplicated environments. This shows that neural-network-based methods cannot sufficiently cope with various environments even if they are trained by using a lot of data.

## 5. Conclusions

We showed that the proposed approach can efficiently extract line segments as lane-marking features and accurately detect lane markings on various road environments by using these features. In an image, the intensity difference along the horizontal direction in a local region of lane markings forms a hat shape and lane-marking widths in each row have different sizes. Therefore, we applied the proposed hat filter with adaptive sizes to each row of the image. Then, pixels with local maximum values are extracted from the filter responses. They are used as the nodes of a connected graph structure and the edges of the graph are constructed by using the proposed neighbor searching method. In the graph, nodes related to lane markings are selected by finding a connected subgraph, and the selected nodes are fitted to line segments for lane markings. The experimental results showed that the proposed method not only yields at least 2.2% better performance compared to the existing methods on the KIST dataset which includes various types of sensing noise caused by environmental changes, but also improves at least 1.4% better than the previous methods on the Caltech dataset which has been widely used for comparison of lane marking detection. Furthermore, the proposed lane marking detection runs with an average time of 3.3 ms, which is fast enough for real-time applications. As for future work, we plan to investigate how virtual lane markings for the host lane can be estimated on a road image without lane markings.

## Figures and Tables

**Table 1:** Nomenclature table for principal components.

Variable	Description
$x_{i},y_{j}$	pixel coordinate on an image
$\gamma_{ij}$	lane-marking score
$p_{k}$	lane-marking feature point
G	graph G = (P,E)
$g^{t}$	lane-marking graph, t-th undirected subgraph of G
$P^{t}$	a set of nodes in *t*-th lane-marking graph
$E^{t}$	a set of edges in *t*-th lane-marking graph
$g_{opt}^{t}$	the connected optimal subgraph, $g_{opt}^{t}\subset g^{t}$

**Table 2:** Lane detection accuracies on KIST dataset [19].

		Day	Tunnel	Rain	Backlight	Night
LSD+VP [36]	Left	85.52	69.29	75.23	53.20	67.68
	Right	80.38	69.70	70.72	53.20	68.44
	Both	82.95	69.50	72.97	53.20	68.06
Canny+HT [35]	Left	81.52	71.20	77.93	83.01	49.43
	Right	68.95	85.33	35.59	56.82	72.62
	Both	75.24	78.26	56.76	69.92	61.03
ST+HT [39]	Left	96.57	**100.00**	97.75	97.75	72.62
	Right	94.29	**99.86**	86.49	68.80	97.72
	Both	95.43	**99.93**	92.12	83.15	85.17
LSD+VPR [19]	Left	97.33	99.59	96.85	88.02	**97.72**
	Right	96.57	99.05	99.55	94.99	99.24
	Both	96.95	99.32	98.20	91.50	98.48
EDLines+KF [20]	Left	50.01	58.70	N/A	24.51	21.67
	Right	43.43	44.84	N/A	22.01	24.33
	Both	46.76	51.77	N/A	23.26	23.00
EDLines+VPR	Left	90.86	99.18	86.94	98.61	**97.72**
	Right	98.10	95.79	96.40	97.21	96.20
	Both	94.48	97.49	91.67	97.91	96.96
LaLi+VPR	Left	**99.05**	99.46	**98.65**	**98.89**	97.34
	Right	**98.29**	99.59	**100.00**	**100.00**	**100.00**
	Both	**98.67**	99.52	**99.32**	**99.44**	**98.67**

**Table 3:** Lane detection accuracies on Caltech dataset [34].

		cordova1	cordova2	washington1	washington2
HT+RANSAC [34]	Left	92.20	89.41	**94.66**	88.79
	Right	92.00	61.33	90.21	96.12
	Both	91.60	75.73	92.43	92.46
LSD+VPR [19]	Left	92.80	90.89	92.28	**98.28**
	Right	96.00	**83.99**	86.94	98.28
	Both	94.40	87.44	89.61	98.28
EDLines+KF [20]	Left	11.60	38.92	37.39	13.79
	Right	16.80	33.99	37.09	11.64
	Both	14.20	36.45	37.24	12.72
EDLines+VPR	Left	91.20	93.38	93.18	94.40
	Right	**97.60**	80.05	**92.58**	96.98
	Both	94.40	86.70	**92.88**	95.69
LaLi+VPR	Left	**95.20**	**97.54**	**94.66**	**98.28**
	Right	**97.60**	79.56	89.61	**98.71**
	Both	**96.40**	**88.55**	92.14	**98.49**

## Data Availability

Not applicable.
